# Exploring key job demands and resources in Norwegian child mental health services: a cross-sectional study of associations with and relationship between compassion satisfaction, burnout, secondary traumatic stress and turnover intention

**DOI:** 10.3389/fpubh.2024.1304345

**Published:** 2024-03-06

**Authors:** Samira Aminihajibashi, Tine K. Jensen, Ane-Marthe Solheim Skar

**Affiliations:** ^1^Norwegian Centre for Violence and Traumatic Stress Studies, Oslo, Norway; ^2^Department of Psychology, University of Oslo, Oslo, Norway

**Keywords:** professional quality of life, occupational health, professional well-being, child therapists, implementation, evidence-based practices, trauma-focused cognitive behavioral therapy, job resources-demands model

## Abstract

**Background:**

Burnout, secondary traumatic stress, and high turnover rates among child mental health clinicians are a challenge, not only for the individual therapist and the organization but also for the successful implementation of evidence-based practices. However, little is known about which and how job-and implementation-related factors are associated with burnout, secondary traumatic stress, and turnover intention as well as compassion satisfaction among child therapists. In the present study, we aimed to explore these factors and related mechanisms by integrating the “professional quality of life” and the “job demands-resources” models of occupational health.

**Methods:**

We measured the perceived professional quality of life and turnover intention among a national sample of 256 therapists working in Norwegian Child and Adolescence Mental Health Clinics (*n* = 44) that implemented Trauma-Focused Cognitive Behavior Therapy (TF-CBT). Seventeen Job-and implementation-related resources and demands were also measured using the General Nordic Questionnaire for Psychological and Social Factors at Work and the Implementation Component Questionnaire. Path analysis was used to test whether burnout and compassion satisfaction mediate the relationship between job demands and resources on one hand, and secondary traumatic stress and turnover intention on the other hand.

**Results and discussion:**

Results revealed that two job resources, i.e., positive challenges at work and mastery of work, were significant predictors of all professional outcomes. The proposed model was only partly supported. That is, while burnout did mediate the relationship between some job demands (i.e., work-family interference and role conflict) and job resources (i.e., human resource primacy, positive challenges, and mastery of work) with secondary traumatic stress and turnover intention, compassion satisfaction did not mediate the relationship between job resources and turnover intention. Moreover, in addition to their indirect effects via burnout, role conflict and organizational climate (human resource primacy) also directly affected turnover intention. These findings propose that interventions that reduce burnout should be prioritized to improve the professional quality of life and turnover intention among child therapists. Theoretically, it seems that compassion satisfaction and work engagement act differently.

## 1 Introduction

Working therapeutically with people with mental health problems can be highly rewarding, but also demanding and draining. Compared to professionals from other healthcare sectors, mental health professionals report poorer well-being (1), and high rates of both burnout (2, 3) and secondary traumatic stress (4, 5). At the same time, they also express a high level of compassion satisfaction [(6, 7); see section 2.2 for definition of these concepts]. Particularly working with traumatized and mentally distressed children has been reported to be emotionally more demanding than working with adults (8–10). If unattended to, the negative aspects of working with traumatized children can cause physical and mental health problems, e.g., anxiety, depression, substance abuse, psychosomatic pains, and sleep disorders (11, 12), and may lead mental health professionals to consider leaving their job. In fact, turnover *intention* rates among child therapists working in Child and Adolescent Mental Health Services (CAMHS) can be as high as 30 to 70% (2, 6), and turnover intention has been found highly correlated with the actual turnover rates in various studies (13, 14). This high turnover is costly and has the potential to adversely impact care quality, continuity, and the sustainability of implemented evidence-based practices (15, 16). A better understanding of the risk and protective factors associated with therapists’ professional quality of life is crucial for early intervention in preventing turnover intention. Such insights hold significant socioeconomic value for both clinicians, decision-makers, and, most importantly, for therapeutic benefits of children in need of mental health services. This knowledge is also essential from an occupational health perspective, because it aligns with the objective of maintaining and promoting the workers’ well-being and work capacities through developing national and institutional action plans (17). This study aims to explore job resources and demands that child mental health institutions can potentially modify to enhance the professional quality of life and retention intention among child therapists. Focusing on work-related factors is favorable as organizational interventions may be more malleable and efficient in improving staff well-being and reducing burnout compared to therapists-related factors, such as age, previous trauma experience, and personality.

Numerous studies have investigated factors associated with therapists’ professional quality of life and turnover intention (7, 10, 18–27). However, there are several caveats in the literature. Firstly, the work-related factors relevant to turnover intention and professional quality of life, particularly those linked with compassion satisfaction among child therapists, remain understudied, despite evidence indicating that work-related predictors are likely to be job-and context-specific (28). Secondly, despite growing interest in implementing evidence-based practices in CAMHS, there is limited knowledge about the impact of implementation components on the professional quality of life and turnover intention among these therapists. Thirdly, the mechanisms though which work- and implementation-related resources and demands influence the components of professional quality of life and turnover intention among child therapists remain unknown. To address these caveats, we use two well-established occupational health models, i.e., the “job demands-resources “(JD-R) model, and the “professional quality of life (ProQOL) model, to explore two research questions: *what* and *how* work-, and implementation-related factors are associated with components of professional quality of life (i.e., compassion satisfaction, burnout, and secondary traumatic stress) and turnover intention. Our study focuses on a national sample of therapists working in Norwegian CAMHS that implement Trauma-Focused Cognitive Behavioral Therapy (TF-CBT) (29), a well-researched and widely recommended evidence-based practice for treating child posttraumatic stress (30). Identifying the specific implementation- and work-related resources and demands in this context and how they influence therapists’ health and decisions will enable the development of tailored strategies to optimize the integration of evidence-based approaches, to enhance the overall well-being and job satisfaction of child therapists, to retain skilled professionals and ultimately to foster a more sustainable work environment in child mental health services.

## 2 Theoretical background and hypothesis development

### 2.1 Job demands-resources theory

The JD-R theory is among the most well-established theories within occupational health (31, 32), and includes both negative and positive predictors and outcomes of workplace well-being and health. According to this theory, a worker’s mental health and well-being depend upon the levels of job demands and job resources. Job demands refer to the physical, psychosocial, and organizational characteristics of a job that require physical or mental effort. Under certain circumstances, when relevant organizational and personal resources are available, *challenging* job demands (e.g., complex tasks) can have a positive effect on personal well-being and organizational performance. H*indering* job demands (e.g., conflicts, sustained workload), however, can initiate a “health impairment” process which is characterized by high levels of effort expenditure and fatigue that can lead to high levels of job strain, burnout, and, eventually, mental and physical health problems along with organizationally withdrawal behaviors like job turnover (28). Job resources, however, refer to the aspects of work (e.g., social support, job control) that can help to deal with job demands and achieve work goals through satisfying human’s basic psychological needs (e.g., relatedness and autonomy) and initiating a “motivational process” that leads to work engagement, defined as a positive and fulfilling work-related state of mind that is characterized by vigor, dedication, and absorption (33), and is linked to low intentions to job turnover (34). The revised JD-R model assumes that burnout (negative strain) and work engagement (positive well-being) mediate the relationship between job demands and job resources on one hand and workers’ mental and physical health, as well as organizational outcomes, on the other hand (28, 32). The revised JD-R theory also incorporates personal resources, such as optimism, self-efficacy and other positive self-evaluations about one’s ability to effectively influence the environment. Such resources have the potential to enhance an employee’s well-being by facilitating access to job resources and mitigating the impact of job demands (35).

### 2.2 Professional quality of life theory

Another well-established theory within occupational health is the ProQOL theory (36) which combines the positive and negative aspects of working with trauma and therapy or other caring professions. According to the ProQOL, *compassion satisfaction*; defined as the feeling of fulfillment and pleasure from helping others and doing a good job, is the potentially positive aspect of caring professions. The potentially negative aspect of helping professions is *compassion fatigue,* which is a controversial topic in the literature and has been defined and referred to by different terms (37). According to the ProQOL theory, compassion fatigue consists of two components known as *burnout* (i.e., feelings of exhaustion, disconnectedness, hopelessness, anger, depression, and reduced perceived self-efficiency) and *secondary traumatic stress, STS* (i.e., symptoms similar to post-traumatic stress disorder like having invasive thoughts, sleep problems and re-experiencing the traumatic events that clients have described in detail) (36, 38). Like the JD-R model, the ProQOL model proposes that the therapists’ professional well-being (compassion satisfaction and burnout) and mental health (secondary traumatic stress) depend on the balance between (work-, patient- and therapist-related) risk and protective factors that contribute to the perceived quality of life. However, the ProQOL model does not propose any mediation pathway.

### 2.3 Testing an integrated JDR-ProQOL model

**Figure 1:**
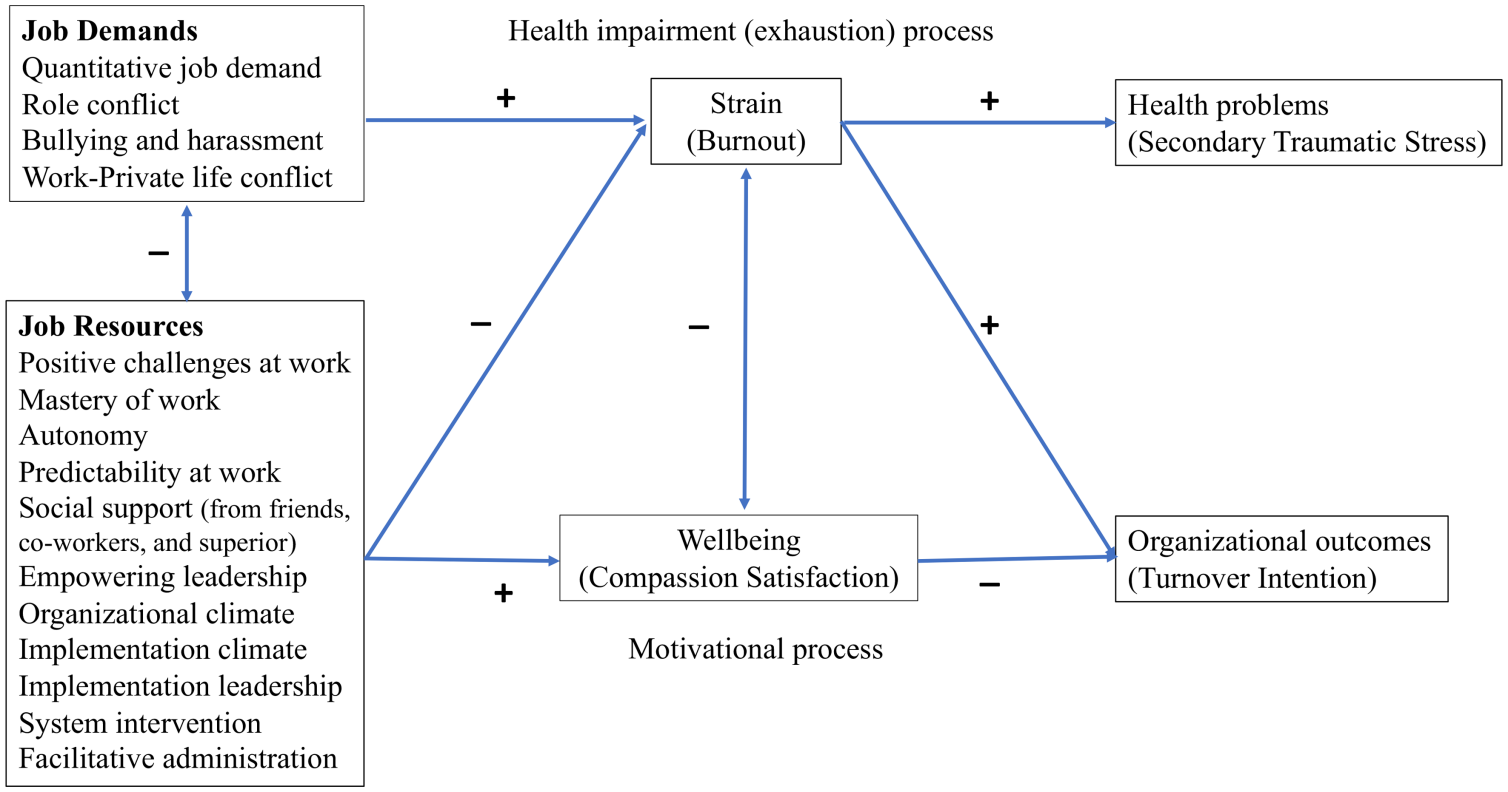
An integrated model of job demands-resources and the professional quality of life theories. Job demands and resources are packed to simplify the illustration and avoid drawing too many paths.

Despite similarities between the ProQOL and the JD-R models, to the best of our knowledge, these two models have not been integrated to investigate which and how job resources and job demands are related to the components of professional quality of life. In fact, a recent review study on work-related factors associated with compassion fatigue in mental health professionals (26) revealed that although findings were consistent with the JD-R model, none of the included studies were theoretically driven. A few studies had used the ProQOL as the theoretical framework to define compassion fatigue, but no study had investigated the job demands and resources associated with compassion fatigue based on the JD-R or other models in occupational health psychology (26). Since both the ProQOL and the JD-R models include the negative as well as positive aspects of (compassionate) work and both propose that personal- and work-related factors have an impact on professional and organizational outcomes, the JD-R model is a plausible framework to explore the relationship between job resources, job demands, components of ProQOL, and the organizational outcomes in a specific job domain and work setting. In this paper, we aim to identify key job demands and resources that contribute to professional quality of life and turnover intention in child mental health professionals using an integrated JD-R–ProQOL model in which burnout and compassion satisfaction would at least partially mediate the effect of job demands and job resources on secondary traumatic stress and turnover intention (see Figure 1).

Professional well-being in the *motivational* pathway of the JD-R model has mainly been measured by work engagement. However, compassion satisfaction could play a similar mediatory role between job resources and organizational outcomes for at least two reasons. Firstly, a strong positive association between compassion satisfaction and work engagement has been found in different professions like teachers (39), police officers (40), residential work care workers (41), and intensive care unit trauma nurses (42). Secondly, empirical findings have shown that they both have a common fostering effect on positive organizational outcomes like decreased turnover intention among health professionals (43, 44). This association and common fostering effect might be related to some shared positive emotions that can broaden an individual’s momentary thought-action repertoire (45), which in turn and over time can promote emotional well-being and build an individual’s intellectual and social skills as well as personal resources. These resources are useful to cope with job demands or achieve job goals by increasing one’s dedication, sense of significance, and enthusiasm (31, 39).

Similarly, in line with the *health impairment* pathway of the JD-R theory, empirical findings have shown that burnout mediates the effect of both the high level of job demands and low levels of job resources on health problems, sick leave, and turnover intention in various job domains (31, 32, 43, 46–48). Therefore, one could expect that the negative effect of working with traumatized children (as a job demand) on developing secondary traumatic stress (as a health problem) is at least partly mediated by burnout (8). Consistent with such an approach, reviewing the literature on compassion fatigue among nurses, Sabo (37) proposed a continuum of occupational stress from burnout to secondary traumatic stress. Kim et al. (49) also found that both compassion satisfaction and burnout mediate the relationship between nurses’ personality type (i.e., a worker-related factor in the ProQOL or a personal resource in the JD-R model) and the negative and positive professional outcomes (i.e., job stress and satisfaction). Finally, Singh et al.’s (26) review study showed that burnout had a moderate to strong relationship with, respectively, compassion satisfaction and secondary traumatic stress in mental health professionals whereas the relationship between compassion satisfaction and secondary traumatic stress was weak. They also found that job demands, i.e., workplace trauma, high caseload of traumatized clients, and challenging therapeutic settings were positively associated with burnout and secondary traumatic stress, whereas job resources, i.e., support from coworkers and supervisors, and organizational support, e.g., trauma-specific training and use of evidence-based practices, were negatively related to burnout and secondary traumatic stress and positively with compassion satisfaction.

#### 2.3.1 Job demands and resources

According to the JD-R theory, *all* and *any* job demand and resource that may have direct or indirect effects on professionals’ well-being, motivation, and strain can be included in the model, although the relevant ones may differ across occupational domains and workplaces (28). There is scarce knowledge about key job demands and resources that are associated with burnout and compassion satisfaction in therapists working in CAMHS. Therefore, in the present study, the job resources and demands are chosen based on previous findings from other workplaces, as well as discussions with field experts to select work- and implementation-related factors that might be relevant to the well-being of child therapists. In the last years, many mental health services acknowledge the value of implementing evidence-based practices such as TF-CBT. In fact, a recent study (6) revealed a lower level of burnout along with a higher level of compassion satisfaction among therapists trained in TF-CBT compared to untrained therapists. However, implementing new treatment programs requires new routines and practices that may put additional demands on mental health professionals (50). In other words, in addition to receiving training in the new programs, other resources (i.e., implementation factors) like having supportive organizations and leaders may be crucial for experiencing compassion satisfaction rather than burnout. Therefore, as also recommended by Bakker and Demerouti (51), job resources and demands at different (individual, team, leader, and organizational) levels are included in the present study and are described below (see also Figure 1).

##### 2.3.1.1 Job demands

High *quantitative job demands*, referring to the amount of work and the associated time pressure; and *role conflicts*, referring to receiving conflicting messages from two or more persons or conflicting and excessive role expectations without sufficient resources, are associated with burnout (52, 53) and turnover intention (54). A recent study of health professionals in Norway (55) found associations between perceived job demands and psychological distress. Similarly, several studies have reported associations between quantitative demands, burnout, secondary traumatic stress, and turnover intention among mental health professionals (26, 56, 57).

*Work-private life conflict* refers to spillover between roles in a way that work-life disturbs private life or the opposite. This is also associated with psychological distress (55), burnout (19, 25), and indirectly with turnover intention (43) in health professionals.

*Bullying and harassment* at the workplace is a hindering factor at the social and organizational level (52), that refers to repeatedly offensive, provoking, or incivility behaviors (e.g., humiliating, gossiping, or withholding information) toward an employee and is associated with poor occupational well-being (58), mental health problems (59), and turnover intention through emotional exhaustion (60).

Based on an integrated JDR-ProQOL model and the reviewed empirical findings, we hypothesized that:

*H1*: Job demands will be positively related to burnout, and burnout will, in turn, be positively related to secondary traumatic stress and turnover intention.

##### 2.3.1.2 Job resources

*Positive challenges at work* and perceived *mastery of work* (61) are associated with low levels of sick leave (62) and compassion satisfaction among child protection workers (63), and with both work engagement, job satisfaction as well as burnout among employees in different sectors including the health sector (52, 64).

*Autonomy* and *predictability at work* make it possible to participate in the process of planning and successful decision-making to provide the best care for the patients. In contrast, uncertainty about roles and responsibilities can be highly demanding (61). In line with this, high levels of predictability and control at work have been found to relate to compassion satisfaction (65), burnout (37), and lower sick leave (62, 66). Job control is also negatively associated with both burnout and turnover intention, and positively with job satisfaction among psychologists (25). In other words, low levels of job resources can also feed into a pathway of health impairment.

*Social support,* including support from family and friends, co-workers, and superiors, are reliable predictors of both burnout and compassion satisfaction among therapists (7, 67), and can mitigate the effect of job demands on compassion fatigue (26) and on secondary traumatic stress (5). The level of positive collegiate support is indeed one of the elements of compassion satisfaction (36).

*Empowering leadership* refers to a participative form of leadership in which the power is shared with the employees and the followers are encouraged to take an active part in decision-making and delegating responsibilities. The purpose is to achieve job goals by enhancing the employee’s intrinsic motivation (e.g., fulfilling the need for self-determination) and self-leadership skills. Empowering leadership can mitigate employees’ levels of perceived stress and contribute to employees’ general mental health (68). Empowering leadership can contribute to compassion satisfaction by fulfilling therapists’ basic psychological need for autonomy and self-efficiency and increasing their sense of involvement in the processes that lead to achieving therapeutic and organizational goals. Engaging leadership has also an indirect effect on burnout and well-being by regulating the existent job resources and demands (69). Empirical findings have shown that empowering leadership has both a direct effect on work engagement and an indirect effect that is partially mediated by other job resources and demands (61, 70).

*Organizational climate,* measured as human resource primacy (HRP), refers to employees’ perception that the organization has an interest in employees’ well-being, health, and welfare, and can be considered a type of organizational support that lowers turnover intentions (71). A recent study among Norwegian child healthcare workers (43) revealed that job autonomy, social support, leadership, and team climate were significant predictors of both work engagement and burnout, which were in turn related to turnover intention. In addition to these work-related resources, some implementation-related resources were included.

*Implementation climate* is similar to organizational climate but is specifically concerned with employees’ perception that the organization supports and facilitates an implementation effort, which is critical for implementation success (72).

*Implementation leadership* refers to the quality of leadership behavior specifically targeting to support and enable an implementation process.

*System interventions* refer to organizational strategies to provide support from other external systems (73, 74).

*Facilitative administration* refers to the amount of organizational restructuring to make implementation and sustainability successful.

To the best of our knowledge, the effect of implementation-related factors on therapists’ professional quality of life has not been studied. However, based on the assumptions of both the JD-R and the ProQOL models, one can expect that an adequate level of implementation-related resources can decrease therapists’ strain and turnover intentions among therapists who have participated in an evidence-based practice program, because access to organizational, administrative, and managerial support can increase therapists’ sense of belonging and commitment and they can better focus on their primary tasks and duties, leading to higher satisfaction derived from helping and serving others. Consistently, some studies have reported a relationship between implementation climate and employees’ burnout and mental health (75), and between organizational support, compassion satisfaction, burnout, and secondary traumatic stress (76). Based on an integrated JDR-ProQOL model and the reviewed empirical findings, we hypothesized that:

*H2*: Job resources will be negatively associated with burnout and positively related to compassion satisfaction and, compassion satisfaction will, in turn, be negatively related to turnover intention. In other words, burnout mediates the effect of job demands and (inadequate) job resources on secondary traumatic stress and turnover intention whereas compassion satisfaction mediates the effect of job resources on the turnover intention at least partially (Figure 1).

These two hypotheses are developed to explore and predict (RQ1): *How* are these work- and implementation-related factors are related to the measured outcomes? As mentioned, we will also seek to explore:

*RQ2*: What are the key job resources and demands associated with the measured outcomes, i.e., burnout, secondary traumatic stress, compassion satisfaction, and turnover intention among child therapists working in CAMHS?

Although in theory, all these job demands and resources can have direct or indirect effects on professional outcomes, in practice, the relevant resources and demands can differ across occupational domains and workplaces (28) and need to be explored. Moreover, since the components of professional quality of life are, theoretically and empirically, independent concepts that are related to each other (36, 77), they can have both component-specific and shared predictors.

## 3 Materials and methods

### 3.1 Procedures and participants

This is a cross-sectional study of mental health professionals working in Norwegian CAMHS, 5 years after an implementing program was started for routine trauma screening procedures followed by TF-CBT for those in need of trauma treatment (i.e., *ca.* sustainment phase of implementation). The implementation program involved training all therapists in trauma and post-traumatic stress screening and training a sub-sample of volunteer therapists in TF-CBT. All therapists and leaders (*N* = 699) working at the time of data collection in one of 44 Norwegian CAMHS in which TF-CBT was implemented were invited to participate. An online questionnaire was sent with two reminders.

A total of 256 therapists (females = 213) responded to all questionnaires, indicating a response rate of 37%. Almost half of the participants (*N* = 116; 45.3%) were psychologists. Other disciplines included medical doctors (6.6%), psychiatrists (2.7%), social educators (17.6%), child welfare officers (12.9%), family therapists (5.5%), nurses (3.5%), and social workers (5.9%). One third (34%) of participants were between 40 to 49 years old (*N* = 88), but there was a broad age span from 20 to more than 60 years. Around 50% (*N* = 124) had worked in the current CAMHS over 6 years. About one-third (*N* = 92; 36%) of the therapists were TF-CBT practitioners, and 46 (18.0%) also had leadership positions. All participants signed a consent form. The study received ethical approval from the Norwegian Regional Committee for Medical and Health Research Ethics (ref. 2017/1619).

### 3.2 Measures

Instead of independent and dependent variable terms, in path analysis, variables are defined as exogenous variables (those that straight arrows emerge from but no arrow points to them), and endogenous variables that have at least one straight arrow pointing to them (78).

#### 3.2.1 Exogenous variables

##### 3.2.1.1 Job demands

The General Nordic Questionnaire for Psychological and Social Factors at Work (QPS_Nordic_ (61)) was used to measure four *hindering* aspects of the workplace that can initiate a “health impairment” process, that is, quantitative job demands, role conflict, work-private life interaction, and bullying and harassment. Examples of the items include “Is it necessary to work at a rapid pace?” (quantitative job demands), “Do you have to do things that you feel should be done differently?” (role conflict), “Do the demands of your work interfere with your home and family life (work-private life interaction), and “have you been subjected to bullying or harassment in the past 6 months?” (bullying and harassment). Participants were asked to indicate their response by using a 5-point Likert rating scale from ‘very seldom or never’ (1) to ‘very often to always’ (5). A mean score was calculated for each subscale. Higher scores indicate a higher level of job demands. The internal consistencies for these scales were satisfactory (see Table 1).

**Table 1:** Descriptive statistics for exogenous and endogenous variables.

Variables	Mean (SD) (SD)	Possible Range	Items (α)	Skewness (Kurtosis)
ICQ Facilitative administration	5.54 (1.27)	1–7	4 (0.71)	−1.3 (1.96)
Implementation leadership	5.31 (1.46)	1–7	9 (0.83)	- 1.08 (0.39)
Systems interventions	5.07 (1.94)	1–7	1	−1.02 (−0.28)
Implementation climate	6.06 (1.04)	1–7	4 (0.75)	−2.05 (5.72)
QPS _Nordic:_ Quantitative job demands	3.83 (0.70)	1–5	4 (0.80)	−0.28 (−0.27)
Bullying and harassment	0.24 (1.06)	0 - n	5 (0.85)	7.86 (74.28)
Work–Family Conflict	3.07 (0.90)	1–5	1	−0.14 (−0.04)
Role conflict	2.61 (0.77)	1–5	3 (0.73)	0.22 (−0.25)
Positive challenges at work	4.35 (0.55)	1–5	3 (0.75)	−1.12 (2.11)
Control of decision	3.47 (0.61)	1–5	5 (0.68)	−0.18 (−0.14)
Predictability at work	4.19 (0.67)	1–5	3 (0.65)	−1.50 (3.71)
Mastery of work	3.98 (0.46)	1–5	4 (0.69)	−0.64 (2.09)
Support from family and friends	3.78 (0.97)	1–5	3 (0.77)	−0.60 (−0.23)
Support from co-workers	4.41 (0.66)	1–5	2 (0.77)	−1.11 (1.12)
Support from leader	3.97 (0.92)	1–5	3 (0.88)	−0.71 (−0.06)
Empowering leadership	3.57 (0.98)	1–5	3 (0.87)	0.44 (−0.18)
Human resource primacy	3.08 (0.92)	1–5	3 (0.81)	0.07 (−0.58)
PROQOL Compassion satisfaction	40.51 (4.95)	10–50	10 (0.85)	−0.26 (−0.28)
Burnout	20.85 (4.51)	10–50	10 (0.74)	0.14 (−0.47)
Secondary traumatic stress	19.39 (4.41)	10–50	10 (0.76)	0.85 (0.16)
Turnover intention	15.34 (4.53)	6–30	6 (0.77)	0.17 (−0.65)

ICQ, Implementation Component Questionnaire; QPS_Nordic_, General Nordic Questionnaire for Psychological and Social Factors at Work; ProQOL, Professional Quality of Life Scale (based on raw scores). α: Cronbach’s alpha. Kurtosis value > 3 may indicate that a variable is not normally distributed (82).

##### 3.2.1.2 Job resources

The QPS _Nordic_ scales were also used to measure nine job resources (see Table 1). Examples of the items include “Are your skills and knowledge useful in your work?” (positive challenges at work), “If there are alternative methods for doing your work, can you choose which method to use?” (control of decisions), “Are you content with the quality of the work you do?” (mastery), “Do you know in advance what kind of tasks to expect a month from now?” (predictability), “If needed, can you talk with your spouse or any other close person about your work-related problems?” (support from family and friends), “If needed, can you get support and help with your work from your immediate superior?” (support from superior), “If needed, can you get support and help with your work from your coworkers?” (support from coworkers), “Does your immediate superior encourage you to participate in important decisions?” (empowering leadership), “To what extent is the management of your organization interested in the health and well-being of the personnel” (human resource primacy). Participants were asked to indicate their response by using a 5-point Likert rating scale from ‘very seldom or never’ (1) to ‘very often to always’ (5). A total score was measured for perceived social support. The internal consistencies for these scales were satisfactory (see Table 1).

Job resources directly related to the ongoing implementation of TF-CBT in the mental health clinics were measured by questions from the Implementation Component Questionnaire (ICQ (73, 74, 79)) measuring facilitative administration, implementation leadership, and systems interventions. Examples include “Administrative practices and procedures have been changed to meet specific needs related to the implementation of TF-CBT” (facilitative administration), “Leaders within the organization have been actively engaged in resolving any and all issues that got in the way of using and implementing TF-CBT effectively” (implementation leadership), and “Administrative staff of the provider organization (key directors, managers, and supervisors) have secured adequate resources to sustain TF-CBT effectively, e.g., assure appropriate referrals, sufficient funding, staff certification” (systems interventions, 1 item). In addition, implementation climate was measured by items adapted from Fixsen et al. (79). An example includes “The implementation of TF-CBT has had positive consequences for my workplace.” Each item had nine response alternatives from ‘completely disagree’ = 1, to ‘completely agree’ = 7, along with ‘neither agree nor disagree’ = 4, ‘I do not know’ = 8, and ‘not relevant’ = 9 response alternatives, where the last two responses were coded as missing, following Ogden et al. (73). Participants were asked to respond based on their evaluation of the situation in the last 6 months. After reversing the scores of some items, mean scores for each subscale were computed for each individual if at least half of the items were answered. Higher scores indicate better implementation-related job resources. All internal consistencies were satisfactory (see Table 1).

#### 3.2.2 Endogenous variables

##### 3.2.2.1 Professional quality of life

Compassion satisfaction, burnout, and secondary traumatic stress were measured by subscales of the Professional Quality of Life Scale, version 5 [ProQOL-V; (36)]. Examples include “I invigorated after working with those I help” (compassion satisfaction), “I feel overwhelmed because my workload seems endless” (burnout), and “I think that I might have been affected by the traumatic stress of those I *help*” (secondary traumatic stress). Respondents are asked to read each statement and determine how frequently they have experienced it in the last 30 days by choosing one of the 5 alternatives from never (1) to very often (5). Following Stamm (36), we converted the sum scores of each subscale to t-scores with a mean of 50 and a standard deviation of 10. This approach has the advantage that scores from different subscales, ProQOL versions, and studies can be comparable. The scales have been found to have good to excellent reliability (Cronbach alpha ranging from 0.75 to 0.88) and good construct validity (36). The Norwegian version of The ProQOL-V (80) was used in the present study.

##### 3.2.2.2 Turnover intention

Turnover intention was measured by the short version of the Turnover Intention Scale (81). Examples of the 6 items include ‘How often have you considered leaving your job?’ and “How likely are you to accept another job at the same compensation level should it be offered to you?.” Participants indicate their response by using a 5-point Likert rating scale from ‘never’ (1) to ‘always’ (5). A sum score (from 6 to 30) was computed for each participant. The scale was translated to Norwegian using the translate-back-translate method. The number of items used to measure each variable and Cronbach’s alphas are presented in Table 1 and show good reliability.

### 3.3 Missing data

Mean scores were computed for each individual if at least half of the items were answered. The sample size varies between variables because of missing data (either due to not responding or coding a response as missing data). There were no missing data in QPS (i.e., 13 exogenous variables) and turnover measurements. Missing data in all subscales of ProQOL was 11% because leaders (*n* = 29) did not respond to this questionnaire, implying missing data at random (MAR). Missing data in ICQ measurements, however, varied across the subscales and only three participants had no data on any subscale (implementation climate = 4.7%, implementation leadership = 6.6%, system intervention = 12%, and facilitative administration = 12%). Missing data were handled using the Full Information Maximum Likelihood method in R statistical software [v4.2.2; (83)].

### 3.4 Statistical analyses

IBM SPSS Statistics [Version 27.0, IBM Corporation (84)] is used to conduct descriptive and bivariate correlation analyses. Path analysis is conducted using R (83), RStudio (85), the “lavaan” package (86), and the JDR-ProQOL model to explore the key predictors of professional quality of life and turnover intention among child therapists and to examine the mediating role of burnout and compassion satisfaction in the relationship between job demands and job resources, and work-related professional outcomes (i.e., secondary traumatic stress and turnover intention). The “Bullying and harassment” variable is not included in the path analysis because there were only 13 participants who reported such an observation or experience and therefore these data were too skewed. Also, since the overall concept of social support was our primary focus, only total scores of perceived social supports were included in the path analysis to increase model parsimony and power of estimations.

Visual inspections along with kurtosis and skewness values (Table 1) indicated that the distribution of all endogenous variables was within the accepted range of univariate normal distributions according to Kline (87). However, since data were not complete and results from Mardia’s multivariate normality test of mvn function (88) showed statistically significant degrees of multivariate non-normality, the Maximum Likelihood Robust (MLR) estimation method is used in the “sem” function (89) to estimate the robust standard errors and to assess overall model fit by corrected chi-square (χ^2^)/degrees of freedom ratio, and other four robust model fit measurements: Goodness-of-Fit statistic (GFI), comparative fit index (CFI), root mean square error of approximation (RMSEA), and Tucker Lewis index (TLI). Recommended values to indicate good model fit is χ^2^/*df* < 3 or < 5, along with GFI, CFI, and TLI values >0.90 or 0.95, and RMSEA <0.06 or.07 (90–92). Since the nature of the study is mainly exploratory, if results from SEM analysis do not reveal acceptable measures across all these model fit indices, the largest modification indices will be considered to respecify the model before testing hypotheses only if the proposed changes align with research questions and theoretically make sense.

Finally, following recommendations (93), the bias-corrected and accelerated (“bca.simple”) bootstrapping option in “parameterEstimates” function (94) is used with 5,000 resampling with replacement to compute confidence intervals and re-evaluate the significance of indirect and total effects. Since results revealed the same conclusions (i.e., coefficients and their statistical significance were comparable) across two methods, standardized parameters estimated by MLR and FIML are reported that were more conservative. Standardized direct effects of 0.1, 0.3, and 0.5, along with standardized total and indirect effects of 0.01, 0.09, and 0.25 are considered small, medium, and large effect sizes, respectively (95). As reported in Table 1, two out of 15 exogenous variables (i.e., work-private life conflict and system intervention) were measured by 1 item with a 5 and 7 Likert scale, respectively. However, there is substantial evidence in the literature that provides support for applying MLR estimation and parametric statistics when having both continuous and ordinal variables, conditioning that the ordinal variable has more than four categories (96–99). Consistently, results from Spearman’s and Pearson’s correlation analysis in the current study revealed similar correlation coefficients between these two variables and the outcome variables. Last but not least, previous simulation studies have revealed that no statistical power analysis can determine a single minimum sample size requirement that is sufficient for evaluating different aspects of model testing, and parameter estimation in path analysis (101). Some commonly used rules of thumb recommend having at least 100 or 200 subjects, 5 observations per estimated parameter, or 10 cases per variable (102). Although our sample size satisfies these minimum requirements, the discussion will acknowledge possible limitations in the interpretation of results as these rules may not consistently align with each other or with outcomes derived from alternative power estimation approaches (102).

## 4 Results

### 4.1 Correlates of professional quality of life and turnover intention

Results from the bivariate correlational analysis are presented in Table 2, showing that, as expected, burnout, secondary traumatic stress, and turnover intention correlated positively with each other and negatively with compassion satisfaction. That is, higher ratings of burnout and secondary traumatic stress were associated with lower compassion satisfaction and higher intention to leave. Results also show that the direction of relationships between job demands, job resources, and the measured positive and negative professional outcomes were consistent with our expectations (H1 and H2). That is, job demands were positively related to burnout, secondary traumatic stress, and turnover intention and negatively to compassion satisfaction, whereas the opposite relationships existed between job resources and these outcome variables, i.e., higher ratings in job resources were associated with higher satisfaction and lower burnout, secondary traumatic stress and turnover intention. However, some associations were very weak, and a few relationships were not significant (Table 2, for correlation among exogenous variables see Supplementary Table S1).

**Table 2:** Pearson’s correlation analysis.

Variables	Compassion Satisfaction	Burnout	Secondary traumatic stress	Turnover intention
Job demands Quantitative job demands	−0.08	0.37**	0.17**	0.25**
Bullying and harassment	−0.01	0.08	0.07	0.19**
Role conflict	−0.21**	0.47**	0.25**	0.51**
Work-family Interference	−0.27**	0.52**	0.31**	0.34**
Job resources Facilitative administration	0.02	−0.02	0.06	0.07
Implementation leadership	0.20**	−0.28**	−0.15**	−0.37**
Systems interventions	0.12*	−0.23**	−0.17**	−0.28**
Implementation climate	0.16**	−0.16**	−0.01	−0.22**
Positive challenge at work	0.57**	−0.38**	−0.15*	−0.36**
Control of decision	0.22**	−0.32**	−0.20**	−0.29**
Predictability at work	0.12*	−19**	−0.15*	−0.32**
Mastery of work	0.43**	−0.51**	−0.27**	−0.41**
Support from family and friends	−0.02	−0.11	−0.06	−0.07
Support from co-workers	0.24**	−0.29**	−0.16**	−0.41**
Support from leader	0.21**	−0.35**	−0.17**	−0.48**
Total social support	0.17**	−0.32**	−0.17**	−0.41**
Empowering leadership	0.17**	−0.30**	−0.15*	−0.38**
Human resource primacy	0.16**	- 0.23**	−0.02	−0.45**
Outcomes compassion satisfaction	1	−0.57**	−0.16**	−0.29**
Burnout	−0.57**	1	0.49**	0.44**
Secondary traumatic stress	−0.16**	0.49**	1	0.16**
Turnover intention	−0.29**	0.44**	0.16**	1

*Significant at the 0.05 level (1-tailed). **Significant at the 0.01 level (1-tailed).

### 4.2 Model and hypothesis testing

**Figure 2:**
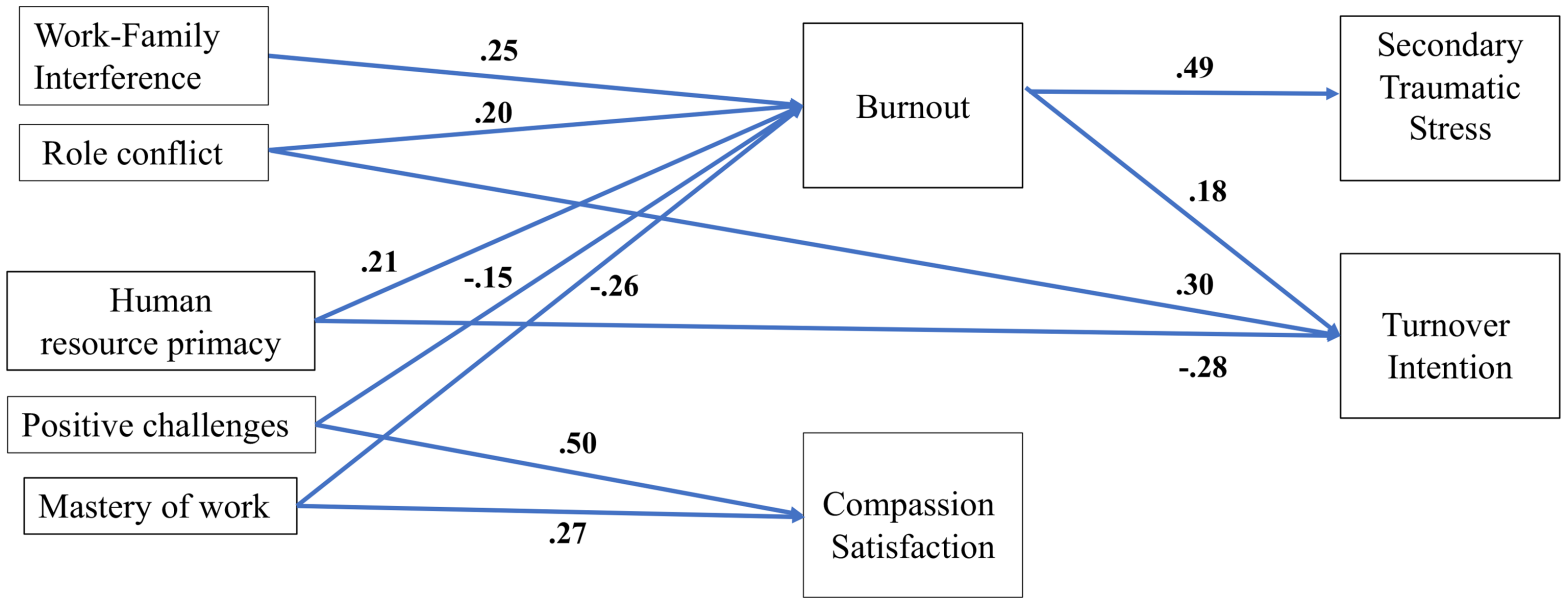
The re-specified model (M2) and the standardized path coefficients. To simplify the illustration and avoid drawing too many paths, only significant paths are included.

We next used the JDR-ProQOL model and conducted path analysis to investigate which, to what extent, and how the work- and implementation-related job demands and resources measured in the present study are related to the professional outcomes (i.e., compassion satisfaction, burnout, secondary traumatic stress, and turnover intention). We started with a full path model (M1), including all variables and pathways in the model (Figure 1). As seen in Table 3, results did not show good model fits across all measurements of goodness of fit (i.e., TLI < 0.90 and RSMEA >0.07). Results from estimating Modification Indices (M.I.) revealed that adding direct paths from human resource primacy and role conflict to turnover intention would improve model fit equally large (M.I. = 39.43 and 38.81, respectively). These proposed model modifications are in line with multilevel frameworks of turnover intention (71, 103) and empirical findings showing that job characteristics, along with social and work-context characteristics including human resource primacy and practices (104) and role conflict (105) can have both direct and indirect effects on turnover intention through other mechanisms than burnout like team psychological safety, self-enhancement, and social exchange processes. It is also in line with our research aims (i.e., exploring key work-related predictors of measured outcomes) and our theoretical model proposing that the mediated effect of job resources on turnover intention can be partial. Thus, we added two direct paths from human resource primacy and role conflict to turnover intention. This re-specified model (M2) fitted well with our data across all model fit indices used in the present study (Table 3). The re-specified model (M2) explained 45% of the variance in burnout, 24% variance in self-reported secondary traumatic stress symptoms, 37% in compassion satisfaction, and 38% in turnover intention. This model was used to investigate our hypotheses and research question. Figure 2 presents the re-specified model (M2), illustrating only the significant paths.

**Table 3:** Path models: goodness of fit statistics.

	*χ* ^2^	*df*	*p*	*χ*^2^/df	AGFI	CFI	RMSEA	TLI
M1	116.92	33	<0.001	3.54	0.99	0.95	0.10	0.77
M2	49.33	31	<0.02	1.59	0.99	0.99	0.05	0.94

*χ*^2^: scaled chi-squared statistic; GFI, Goodness of Fit Index; CFI, Comparative Fit Index; RMSEA, Root Mean Square Error of Approximation; TLI, Tucker Lewis Index. All measures are Robust estimations.

*H1*: Job demands will be positively related to burnout, and burnout will, in turn, be positively related to secondary traumatic stress and turnover intention.

Table 4 presents the standardized regression coefficients. As seen, consistent with the JDR-ProQOL model and H1, job demands were positively related to burnout, which was, in turn, positively related to secondary traumatic stress [B_std_ = 0.49, CI (0.38, 0.59), *p* < 0.001], and turnover intention [B_std_ = 0.18, CI (0.04, 0.32), *p* = 0.01]. However, only measures of two job demands (i.e., role conflict and work-private life conflict) contributed significantly to predict the burnout ratings [quantitative job demand did not reach significance level; B_std_ = 0.10, CI (−0.03, 0.23), *p* = 0.13].

**Table 4:** Standardized effects of exogenous variables on measured outcomes.

	Direct effect			Indirect effect		
Variables	Compassion satisfaction	Burnout	Turnover intention	Turnover intention via Comp. Sat.	Turnover intention via burnout	Secondary traumatic stress via burnout
Job demands quantitative job demands		0,10			0,02	0,05
Role conflict		**0,20	**0,30		** 0,04	*** 0,10
Work-family interference		**0,25			** 0,04	*** 0,12
Job resources positive challenges	** 0,50	**- 0,15		−0.04	* −0,03	**- 0,07
Control of decision	0,01	-0,03		−0.01	−0,01	- 0,02
Predictability at work	−0,07	0,04		0.01	0,01	0,02
Mastery of work	** 0,27	**- 0,26		−0.02	** − 0,05	***- 0,13
Total social support	* − 0,11	−0,06		0.01	-0,01	- 0,03
Empowering leadership	0,03	-0,14		−0.003	−0,02	−0,07
Human resource primacy	- 0,09	**0,21	**- 0,28	0.01	* 0,04	** 0,10
Implementation climate	−0,04	0,03		0.003	0,01	0,01
Implementation leadership	0,05	- 0,04		−0.01	−0,01	−0,02
Systems interventions	−0,05	0,03		0.004	0,02	0,02
Facilitative administration	0,10	−0,02		−0.01	−0,001	−0,002
ProQOL compassion satisfaction			−0,09			
Burnout			**0,18			

1. *p < 0.1; **p < 0.05; ***p < 0.01. 2. Standardized direct effects of 0.1, 0.3, and 0.5, along with standardized total and indirect effects of 0.01, 0.09, and 0.25 are considered small, medium, and large effect sizes, respectively (95).

*H2*: Job resources will be negatively associated with burnout and positively related to compassion satisfaction and, compassion satisfaction will, in turn, be negatively related to turnover intention. In other words, burnout mediates the effect of job demands and (inadequate) job resources on secondary traumatic stress and turnover intention whereas compassion satisfaction mediates the effect of job resources on the turnover intention at least partially.

As presented in Table 4 and Figure 2, H2 was just partly supported. That is, firstly, in contrast to H2, one of the job resources (i.e., human resource primacy) was positively related to burnout [B_std_ = 0.21, CI (0.05, 0.37), *p* = 0.009]. Secondly, compassion satisfaction did not show a significant effect on turnover intention [B_std_ = − 0.09, CI (−0.22, 0.05), *p* = 0.20]. In other words, only burnout significantly mediated the effect of job demands and resources on secondary traumatic stress and turnover intention. In fact, only a few job resources were significant predictors of compassion satisfaction and burnout (see next section). Thirdly, one of the job demands (i.e., role conflict), and one the of job resources (i.e., human resource primacy) had also a direct effect on turnover intention. That is, burnout partially mediated the relation between these two factors and turnover intention.

*RQ2*: What are the key job resources and demands associated with the measured outcomes, i.e., burnout, secondary traumatic stress, compassion satisfaction, and turnover intention among child therapists working in CAMHS?

As shown in Table 4 and Figure 2, two out of 11 measured job resources were shown to be significant predictors of compassion satisfaction among respondent child therapists working in Norwegian CAMHS. Positive challenges at work had the strongest effect (B_std_ = 0.50), followed by perceived mastery of work (B_std_ = 0.27). Perceived social support [B_std_ = −0.11, CI (−0.24, 0.02)] and facilitative administration [B_std_ = 0.10, CI (−0.04, 0.23)] did not reach the significant level (*p* = 0.1).

Similarly, three job resources along with two out of three job demands contributed significantly to predicting burnout ratings. Perceived mastery of work (B_std_ = −0.26) and interference between work demands and private life (B_std_ = 0.25) had the strongest effect followed by human resource primacy (B_std_ = 0.21), role conflict (B_std_ = 0.20), and positive challenges at work (B_std_ = −0.15). Empowering leadership [B_std_ = −0.14, CI (−0.30, 0.03)], and quantitative job demand [B_std_ = 0.10, CI (−0.03, 0.23)] did not reach significance level (*p* = 0.1).

Burnout was the strongest predictor of secondary traumatic stress (B_std_ = 0.49). The largest significant indirect effects on secondary traumatic stress were from perceived mastery of work (B_std_ = −0.13), and interference between work and private life (B_std_ = 0.12), followed by human resource primacy (B_std_ = 0.10), role conflict (B_std_ = 0.10), and positive challenges at work (B_std_ = −0.07), all via burnout. Except for human resource primacy and positive challenges at work that did not reach the significance level (*p* = 0.08), the same variables had also significant indirect effects on turnover intention via burnout, although to a smaller extent than their effects on secondary traumatic stress (Table 4). That is, with one standard deviation increase in perceived mastery of work and positive challenges at work, along with one standard deviation decrease in work-life interference and role conflict, turnover intention is predicted to decrease by in sum 0.26 standard deviations via their effects on burnout. However, as mentioned, role conflict and human resource primacy had also a significant direct effect on turnover intention, which were stronger than the effect of burnout on turnover intention (B_std_ = 0.30, −0.28, 0.18, respectively). Total effects of role conflict (0.34), human resource primacy (0.23), positive challenges (−. 07), and mastery of work (−. 07) on turnover intention were significant. Finally, mastery of work was the only factor that had either a significant direct or indirect effect on both compassion satisfaction and negative outcomes (i.e., burnout, secondary traumatic stress, turnover intention).

## 5 Discussion

Studies generally find a high prevalence of burnout and turnover intention among child therapists [e.g., (2, 6)], but also compassion satisfaction (6), with the latter study finding lower level of burnout along with a higher level of compassion satisfaction among therapists trained in an evidence-based practice (TF-CBT) compared to untrained therapists. These and similar alarming prevalence data indicate that more research and more tailored organizational interventions are required to improve child therapists’ occupational health and prevent negative organizational outcomes. In the present study, through integrating the JD-R and ProQOL models of occupational health, we aimed to contribute to the literature by measuring implementation- and work-related resources and demands at different levels and exploring the key predictors and the mechanism by which they are associated with compassion satisfaction, burnout, secondary traumatic stress, and turnover intention among a national sample of therapists working in Norwegian CAMHS.

### 5.1 What are the key predictors of the professional quality of life and turnover intention in child therapists?

Bivariate analysis showed that almost all measured job resources and demands had a significant relationship with the measured outcomes in the expected direction. However, some resources like facilitative administration and support from family and friends did not correlate with any of the measured outcomes, and the bullying and harassment factor was only significantly associated with higher turnover intention. Since only 13 respondents reported experiencing or observing bullying and harassment at the workplace, we did not include this factor in the path analysis. However, some findings indicate that workplace bullying fosters turnover intention and actual turnover behavior through emotional exhaustion mechanisms (106). The correlation between bullying and turnover intention can also indicate that bullying and turnover are both behavioral outcomes of bad job design with unbalanced job demands and resources (107). Given that work interference with home and family was one of the key predictors of burnout among respondents, it was unexpected that support from family and friends was not associated with lower degrees of negative outcomes. However, it is consistent with findings from some other studies showing that only workplace-related social support contributed to predicting burnout among clinicians (108). This might indicate that family support might not be very relevant in work matters, although cultural differences might also play a role (109).

Moreover, although higher quantitative job demands were associated with higher burnout, secondary traumatic stress, and turnover intentions, path analysis revealed that in contrast to role conflict and work-private life conflict, quantitative job demands, that is the amount and the pace of work, were not significant predictors of measured outcomes. A recent study among social service workers in Poland (110) revealed that challenging demands like quantitative demands, compared to hindering demands like role conflict, are only associated with the “exhaustion” component of burnout, but not with the “disengagement from work” component. In fact, although quantitative demands can be challenging, they can also promote a sense of personal and professional growth and increase income. Similarly, although job autonomy and predictability at work were significantly associated with all measured outcomes, in line with the study by Kim et al. (49), they did not protect against therapist emotional exhaustion.

Finally, the unexpected lack of association between facilitative administration and measured outcomes might be related to limited variance in ratings. In fact, most respondents (85%) reported that administrative practices and procedures have been changed to meet implementation-specific needs. The same observation was also found to a lesser extent for other implementation components (i.e., Systems interventions, Implementation leadership, and Implementation climate), and despite significant correlations, these implementation-related resources did not contribute significantly to the variance of measured outcomes. Our findings are similar to findings in Ogden et al.’s study (73) in which the mean scores were all at the upper end of the scale indicating that respondents were very satisfied with these aspects of the implementation programs and that the new demands related to adopting TF-CBT and trauma screening practices were met. Also, a recent study in Norwegian CAMHS revealed that most therapists had highly positive attitudes toward evidence-based practices programs and those trained in TF-CBT reported lower levels of burnout and turnover intention along with a higher level of compassion satisfaction compared to fellows (6).

#### 5.1.1 Key job demands

The results revealed two job demands, i.e., interference between work demands and private life and role conflict as key work-related predictors of the measured negative outcomes, which is consistent with findings from previous studies on healthcare professionals (19, 25, 43, 53–55, 111, 112). They were also negatively associated with compassion satisfaction. These results indicate that receiving conflicting messages and excessive expectations, particularly without sufficient resources and the possibility to do things differently, can have drastic effects on both employees who will not be able to fulfill all expectations simultaneously and mental health care institutions that are struggling with unwanted turnover. According to the integrated JDR-ProQOL model, chronic exposure to these job demands increases the cognitive load and mental effort to constantly navigate and manage conflicting demands and roles which is emotionally taxing and exhausts employees’ mental and physical resources, leading to ineffective detachment from work, more susceptibility to the emotional impacts of their clients’ traumas and developing secondary traumatic stress. Feeling drained and incompetent, along with struggling to have achievement in one’s job, will eventually end in a distant attitude toward the job and the willingness to leave the job. To reduce the impact of role conflict and work-private life conflict, factors such as balancing quantitative demands, providing support, job autonomy and mindfulness practices might be considered on an organizational level [e.g., (50, 113, 114)].

Notably, role conflict also had a direct effect on turnover intention that was larger than its indirect effect through burnout, suggesting that role conflict can lead to turnover intention without feeling burned out. This is in line with multilevel frameworks of turnover intention (71, 103) and empirical findings showing that job characteristics e.g., role conflict (105, 115), along with social and work-context characteristics [e.g., human resource practices (104)], can have both direct and indirect effects on turnover intention through mechanisms beyond burnout such as team psychological safety, self-enhancement, and social exchange processes. For example, according to Organizational Support Theory, human resource practices and job conditions (e.g., role conflict) are among antecedents of employees’ perceived organizational support. Similar to perceived human resource primacy, this perception reflects a general sense of how much the organization values employees’ contributions and cares about their well-being (104). Through initiating a social exchange process, such perceived organizational support will enhance job satisfaction and reduce turnover intention. It is also noteworthy that a recent study found a link between role overload and job strain through enhancing work–family conflict (116). In fact, role conflict was moderately and positively correlated with work–family conflict in the present study too (Supplementary Table S1). The emotional strain from dealing with conflicting roles can spill over to home, as therapists may feel compelled to allocate more time to address each expectation or demand and making it harder for therapists to find a work-life balance. Future studies should explore and compare different mechanisms through which role conflict can lead to turnover intention.

#### 5.1.2 Key job resources

The results also revealed three job resources, i.e., the perceived mastery of work, positive challenges, and human resource primacy, as key work-related predictors of the measured outcomes. Among these, the perceived mastery of work and positive challenges at work were the only factors that were associated with all measured outcomes and their indirect effects on turnover intention and secondary traumatic stress were generally comparable with their direct effects on compassion satisfaction and burnout (considering that indirect effects are multiplication of direct effects (95)). However, all indirect effects were mediated via burnout (see next section). For child therapists, the emotional toll of dealing with traumatized children can be immense, leading to secondary traumatic stress. These job resources can interrupt the health impairment mechanism by bolstering therapists’ resilience and ability to cope with demands. When therapists are content with the quality of the work they do and they think that their skills and knowledge are useful in their work, they are more likely to feel energized and engaged with their work, rather than feeling helplessness and hopelessness reducing the risk of burnout and willingness to leave their jobs. A meta-analysis of 84 quantitative studies revealed that workplace resources at either of the four individual, group, leader, or organizational levels can improve employee well-being and organizational performance equally (117). However, in the present study, the most effective resources (i.e., perceived mastery of work and positive challenges at work) were both at the individual level. Consistent with the present findings, a recent study revealed positive associations between positive work challenges and mastery at work and work-related well-being among nurse leaders in Finland (118). Similarly, a meta-analysis of 183 independent samples revealed that positive challenges at work had a direct effect on job strain and an indirect effect on turnover intention (115). Our results indicate that learning evidence-based therapeutic methods and diving deeper into their professional knowledge can both boost therapists’ compassion satisfaction and willingness to stay at work and reduce mental health distress.

In contrast to H2 and some previous findings (119), human resource primacy was positively related to burnout. Rather than referring to causal effects, this positive relation more probably indicates that therapists who reported a higher degree of burnout also reported having received more support from the organization and acknowledged that the organization cares about the well-being of the personnel. This interpretation is also supported by our other result showing a direct effect of human resource primacy on reduced turnover intention. Similarly, Kruzich et al. (71) found a protective influence of human resource primacy on turnover intention among American public child welfare workers, mediated by the impact of team psychological safety.

### 5.2 How the work-, and implementation-related factors do affect the professional quality of life and turnover intention in child therapists?

The final revised model explained 45% of the variance in burnout, 24% variance in secondary traumatic stress, 37% in compassion satisfaction, and 38% in turnover intention. Overall, the integrated JDR-ProQOL model was partially supported by the results. That is, job demands positively, and job resources negatively were related to burnout, and burnout mediated the relationship between relevant job demands and job resources with secondary traumatic stress and turnover intention through health impairment processes. However, there were two exceptions. That is, one of the job demands (i.e., role conflict), and one of the job resources (i.e., human resource primacy) had also a direct effect on the turnover intention that both were stronger than their indirect effects and also larger than burnout effect on turnover intention. That is, burnout partially mediated the relation between these two factors and turnover intention. In other words, negative perceived organizational climate (human resource primacy) and role conflict can lead child therapists to consider leaving their workplace both via and independently from feeling burnout.

Moreover, consistent with the JDR-ProQOL model, correlation analysis showed that job resources positively and job demands negatively were related to compassion satisfaction, and a lower level of compassion satisfaction was associated with higher risks of burnout and turnover intention.

Overall, the proposed JDR-ProQOL model was just partially supported by the results. That is, higher risks of burnout were associated with higher risks of secondary traumatic stress, and they both were associated with lower compassion satisfaction and higher risks of intention to leave. Moreover, job demands positively, and job resources negatively were related to burnout, and consistent with the proposed JDR-ProQOL model, burnout did mediate the relationship between relevant job demands and job resources with secondary traumatic stress and turnover intention, through health impairment processes. However, there were two exceptions. That is, one of the job demands (i.e., role conflict), and one of the job resources (i.e., human resource primacy) had also a direct effect on the turnover intention that both were stronger than the total effect of burnout on turnover intention. That is, burnout partially mediated the relation between these two factors and turnover intention.

Moreover, although compassion satisfaction was negatively and moderately correlated with turnover intention, in contrast to our hypothesis, when all other relevant variables were held constant, compassion satisfaction did not have a significant effect and did not mediate the effect of job resources on turnover intention. These results imply that consistent with the assumption of the JD-R model, job resources either directly or through their effect on burnout (health impairment mechanism) and work engagement (which is not measured here) affect therapists’ intention to stay at their current job. Findings also support that the exhaustion (health impairment) and motivation pathways are two independent mechanisms and the health impairment mechanism has a heavier weight in explaining the negative outcomes. A recent study with Norwegian health professionals (43) revealed that work engagement affects job satisfaction positively and turnover intention negatively but the strongest relationship between both job demands and job resources and the outcome variables was mediated by burnout.

### 5.3 Theoretical and practical implications

Theoretically, the present results suggest that although compassion satisfaction can be associated with work engagement, as also demonstrated in other studies (39, 41, 42), they are different constructs that impact different organizational outcomes or act through different mechanisms. That is, although compassion satisfaction is important for therapists’ well-being, it does not mediate the effect of job resources on organizational outcomes through the same motivational mechanism that the JD-R model proposes for work engagement. Compassion satisfaction might be a personal resource that mitigates the effect of other resources and demands on occupational health and organizational outcomes, like psychological capital (120) or the meaning of work (121). For example, Tremblay and Messervey (122) found that compassion satisfaction partially moderated the connection between some role overload and job strain among military chaplains. However, compassion satisfaction did not mitigate the relationship between other three job demands (i.e., insufficiency, ambiguity and conflict) and job strain. Future studies should investigate the role and mechanism by which compassion satisfaction modulates professional gains and losses. The health impairment pathway, however, was supported by the present data.

The present study has several practical implications. First, as work-related risk and protective factors are more amenable to change compared to therapist-related factors (such as age, previous trauma experience, tenure, and personality), the current findings hold practical implications for implementing real policy changes and interventions in human resource management within healthcare settings. Specifically, interventions aimed at reducing role conflict, enhancing human resource primacy, and promoting work-life balance are likely to be effective in preventing burnout and turnover intention among child therapists. Second, since perceived positive challenges at work and mastery of work were the only factors that had either a direct or indirect significant effect on all positive (compassion satisfaction) and negative outcomes (burnout, secondary traumatic stress, turnover intention), our findings propose that training interventions like evidence-based practice implementation initiatives can be effective strategies with important implications both from occupational health and organizational perspectives. Third our findings imply that when evidence-based practice programs are, in general, implemented properly and satisfactorily, they would not be accompanied by an increased level of burnout or turnover intention among child therapists.

### 5.4 Study strengths and limitations

The present study replicates some previous findings and expands the literature in three areas. First, to the best of our knowledge, it is the first study that included job resources and demands at different (individual, team, leader, and organizational) levels to reveal the key work-related factors associated with each and all components of professional quality of life and turnover intention among child therapists. Second, to the best of our knowledge, it presents the first data on the implementation-related factors relevant to all components of professional quality of life, and turnover intention among child therapists. Given that there has been a growing interest in implementing evidence-based practices, and empirical findings showing the alarming professional quality of life and an intent to leave the job, knowledge about key implementation components relevant to a higher level of compassion satisfaction and lower risk of burnout, secondary traumatic stress and turnover intention observed among child therapists are highly important to the sustainability of evidence-based practice implementation initiatives. Third, it is the first study that integrates two well-established occupational health models, i.e., the JD-R model, and the ProQOL model, to investigate the mechanisms by which the work- and implementation-related resources and demands affect child therapists’ professional quality of life and turnover intention, and showed that although compassion satisfaction was related to turnover intention it did not mediate the effects of measured job resources on turnover intention. Moreover, the study has sufficient statistical power.

However, there are also limitations that should be kept in mind when interpreting the results. First, since all constructs were measured by self-report questionnaires, there is a risk of common method bias which can lead to spurious correlations among the constructs due to the measurement instruments. For example, some studies have used multifaceted measures including pupil diameter data to track the level of work engagement as a function of mental fatigue while performing cognitve experiments (123). Nevertheless, using these self-reports was more affordable and appropriate than other methods, such as experiments, observation or interviews, because they allowed measuring the variables of interest, i.e., perceived job resources, job demands, professional quality of life, and turnover intention, with well-established, valid scales that have good reliability while protecting participants’ anonymity (124). Future studies can however use different scales to operationalize burnout and secondary traumatic stress constructs to strengthen the concurrent validity of measures.

Second, the cross-sectional design of this study limits causality interpretations. Cross-sectional studies might show stronger relationships between workplace resources, staff well-being, and organizational performance than longitudinal studies (117). Path analysis can evaluate causal hypotheses, but it cannot determine the direction of causality (125). Therefore, before making any firm conclusions, these findings should be replicated, preferably in larger samples. We used path analysis to investigate the proposed hypothesis, which holds the strength to study both direct and indirect effects simultaneously with several variables and allows us to compare the relationship between the variables. However, given that we re-specified the model based on data-driven modification indices, the risk of overfitting might limit the generalizability of our findings. Yet, the proposed paths were empirically and theoretically justified. Moreover, path coefficients and other parameters of a non-fitting model are also unlikely to be replicated because such a model would not describe the properties of the covariance structure in the population. Therefore, since our study and the integrated JDR-ProQOL model were exploratory, and the added direct effects on turnover intention were theoretically reasonable, we found it justified to respecify the model and provide organizationally valuable knowledge on risk and protective predictors of turnover intention among child therapists. Nevertheless, these findings are the first steps and should be replicated in future studies before making firm conclusions. An alternative analytic approach would be to estimate latent factors within an SEM framework. However, with a sample size of 256 respondents, the power to specify models with several latent factors and multiple pathways is likely to be limited. Moreover, by using observed variables in a path analysis we could examine which, to what extent, and how each of job demands and resources are related to measures of compassion satisfaction, burnout, secondary traumatic stress, and turnover intention, and to explore their shared predictors.

Finally, it is also worth mentioning that a respondent rate of 37% is not optimal which is a common challenge and comparable with the rate of participation among mental health therapists (126). However, a 37% full response rate is within the acceptable cutoff for person-level missingness, and it is expected to produce a small underestimation of the relationships among constructs (127). Moreover, according to the registered data (128), our sample demographics are representative of professionals working in Norwegian CAMHS. Nevertheless, it should be considered that other interventions might be required for individuals with a highly poor professional quality of life who, expectedly, may not be motivated to participate.

### 5.5 Conclusion

Our results revealed that two job demands, i.e., interference between work demands and private life and role conflict, and three job resources, i.e., the perceived mastery of work, positive challenges, and human resource primacy as key work-related predictors of the professional quality of life and turnover intention among child therapists working in Norwegian CAMHS. Among these, the perceived mastery of work and positive challenges at work were the only factors that were associated with all measured outcomes. Training programs, like evidence-based practice implementation programs, that can enhance therapists’ skill and mastery of work, and make work challenges to be perceived positively, along with clear task and role descriptions are among interventions that can be considered at both organization and leader levels. These factors can also play a preventive role in the interference between work and private life. Regular screening and individual conversations can help with better-tailored solutions that each staff might need. This suggests the importance of creating work environments where therapists feel empowered and competent and are given opportunities for positive professional growth.

## Data availability statement

The raw data supporting the conclusions of this article will be made available by the authors, without undue reservation.

## Ethics statement

The studies involving humans were approved by Norwegian Regional Committee for Medical and Health Research Ethics (ref. 2017/1619). The studies were conducted in accordance with the local legislation and institutional requirements. The participants provided their written informed consent to participate in this study.

## Author contributions

SA: Conceptualization, Data curation, Formal analysis, Writing – original draft, Writing – review & editing, Methodology, Investigation, Software, Visualization. TJ: Writing – review & editing, Conceptualization, Funding acquisition. A-MS: Funding acquisition, Project administration, Writing – review & editing, Conceptualization.

## References

[1] Johnson J Hall LH Berzins K Baker J Melling K Thompson C. Mental healthcare staff well-being and burnout: a narrative review of trends, causes, implications, and recommendations for future interventions. Int J Ment Health Nurs. (2018) 27:20–32. doi: 10.1111/inm.12416, PMID: 29243348

[2] McNicholas F Sharma S Oconnor C Barrett E. Burnout in consultants in child and adolescent mental health services (CAMHS) in Ireland: a cross-sectional study. BMJ Open. (2020) 10:e030354. doi: 10.1136/bmjopen-2019-030354, PMID: 31959602 PMC7045151

[3] O’Connor K Neff DM Pitman S. Burnout in mental health professionals: a systematic review and meta-analysis of prevalence and determinants. Eur Psychiatry. (2018) 53:74–99. doi: 10.1016/j.eurpsy.2018.06.003, PMID: 29957371

[4] Diehm RM Mankowitz NN King RM. Secondary traumatic stress in Australian psychologists: individual risk and protective factors. Traumatology. (2019) 25:196–202. doi: 10.1037/trm0000181, PMID: 33192712

[5] Sage C Brooks SK Greenberg N. Factors associated with type II trauma in occupational groups working with traumatised children: a systematic review. J. Ment. Health. (2018) 27:457–67. doi: 10.1080/09638237.2017.1370630, PMID: 28898109

[6] Aminihajibashi S Skar AMS Jensen TK. Professional wellbeing and turnover intention among child therapists: a comparison between therapists trained and untrained in trauma-focused cognitive behavioral therapy. BMC Health Serv Res. (2022) 22:1328. doi: 10.1186/s12913-022-08670-3, PMID: 36348429 PMC9644513

[7] Sodeke-Gregson EA Holttum S Billings J. Compassion satisfaction, burnout, and secondary traumatic stress in UK therapists who work with adult trauma clients. Eur J Psychotraumatol. (2013) 4:869. doi: 10.3402/ejpt.v4i0.21869 PMC3877781 24386550

[8] De Figueiredo S Yetwin A Sherer S Radzik M Iverson E. A cross-disciplinary comparison of perceptions of compassion fatigue and satisfaction among service providers of highly traumatized children and adolescents. Traumatology. (2014) 20:286–95. doi: 10.1037/h0099833

[9] Korkeila JA Töyry S Kumpulainen K Toivola JM Räsänen K Kalimo R. Burnout and self-perceived health among Finnish psychiatrists and child psychiatrists: a national survey. Scand J Public Health. (2003) 31:85–91. doi: 10.1080/14034940210133880, PMID: 12745757

[10] Sprang G Clark JJ Whitt-Woosley A. Compassion fatigue, compassion satisfaction, and burnout: factors impacting a Professional's quality of life. J Loss Trauma. (2007) 12:259–80. doi: 10.1080/15325020701238093, PMID: 38200910

[11] Morse G Salyers MP Rollins AL Monroe-DeVita M Pfahler C. Burnout in mental health services: a review of the problem and its remediation. Admin Pol Ment Health. (2012) 39:341–52. doi: 10.1007/s10488-011-0352-1, PMID: 21533847 PMC3156844

[12] Salyers MP Fukui S Rollins AL Firmin R Gearhart T Noll JP. Burnout and self-reported quality of care in community mental health. Admin Pol Ment Health. (2015) 42:61–9. doi: 10.1007/s10488-014-0544-6, PMID: 24659446 PMC4171274

[13] Aarons GA Sommerfeld DH Hecht DB Silovsky JF Chaffin MJ. The impact of evidence-based practice implementation and fidelity monitoring on staff turnover: evidence for a protective effect. J Consult Clin Psychol. (2009) 77:270–80. doi: 10.1037/a0013223, PMID: 19309186 PMC2742697

[14] Ki J Choi-Kwon S. Health problems, turnover intention, and actual turnover among shift work female nurses: analyzing data from a prospective longitudinal study. PLoS One. (2022) 17:e0270958. doi: 10.1371/journal.pone.0270958, PMID: 35802575 PMC9269367

[15] Aarons GA Hurlburt M Horwitz SM. Advancing a conceptual model of evidence-based practice implementation in public service sectors. Admin Pol Ment Health. (2011) 38:4–23. doi: 10.1007/s10488-010-0327-7, PMID: 21197565 PMC3025110

[16] Swain K Whitley R McHugo GJ Drake RE. The sustainability of evidence-based practices in routine mental health agencies. Community Ment Health J. (2010) 46:119–29. doi: 10.1007/s10597-009-9202-y, PMID: 19544094

[17] World Health Organization. *Occupational health*. (2023). Available at: https://www.who.int/health-topics/occupational-health (Accessed February 8, 2023).

[18] Cavanagh N Cockett G Heinrich C Doig L Fiest K Guichon JR. Compassion fatigue in healthcare providers: a systematic review and meta-analysis. Nurs Ethics. (2020) 27:639–65. doi: 10.1177/0969733019889400, PMID: 31829113

[19] Cetrano G Tedeschi F Rabbi L Gosetti G Lora A Lamonaca D. How are compassion fatigue, burnout, and compassion satisfaction affected by quality of working life? Findings from a survey of mental health staff in Italy. BMC Health Serv Res. (2017) 17:755. doi: 10.1186/s12913-017-2726-x, PMID: 29162095 PMC5696765

[20] Craig CD Sprang G. Compassion satisfaction, compassion fatigue, and burnout in a national sample of trauma treatment therapists. Anxiety Stress Coping. (2010) 23:319–39. doi: 10.1080/10615800903085818, PMID: 19590994

[21] Griffeth RW Hom PW Gaertner S. A Meta-analysis of antecedents and correlates of employee turnover: update, moderator tests, and research implications for the next millennium. J Manag. (2000) 26:463–88. doi: 10.1177/014920630002600305

[22] Köverová M. *Predictors of compassion satisfaction and compassion fatigue among helping professionals in Slovakia*. Conference: International psychological applications conference and trends (InPACT) at: Porto, Portugal. (2018).

[23] Ray SL Wong C White D Heaslip K. Compassion satisfaction, compassion fatigue, work life conditions, and burnout among frontline mental health care professionals. Traumatology. (2013) 19:255–67. doi: 10.1177/1534765612471144

[24] Rossi A Cetrano G Pertile R Rabbi L Donisi V Grigoletti L. Burnout, compassion fatigue, and compassion satisfaction among staff in community-based mental health services. Psychiatry Res. (2012) 200:933–8. doi: 10.1016/j.psychres.2012.07.029, PMID: 22951335

[25] Scanlan JN Still M. Relationships between burnout, turnover intention, job satisfaction, job demands and job resources for mental health personnel in an Australian mental health service. BMC Health Serv Res. (2019) 19:62. doi: 10.1186/s12913-018-3841-z 30674314 PMC6343271

[26] Singh J Karanika-Murray M Baguley T Hudson J. A systematic review of job demands and resources associated with compassion fatigue in mental health professionals. Int J Environ Res Public Health. (2020) 17:6987. doi: 10.3390/ijerph17196987, PMID: 32987798 PMC7579573

[27] Turgoose D Maddox L. Predictors of compassion fatigue in mental health professionals: a narrative review. Traumatology. (2017) 23:172–85. doi: 10.1037/trm0000116

[28] Schaufeli WB Taris TW. A critical review of the job demands-resources model: implications for improving work and health In: Bauer GF Hämmig O, editors. Bridging occupational, organizational and public health. Berlin: Springer Science + Business Media (2014). 43–68.

[29] Cohen J Mannarino AP. Disseminating and implementing trauma-focused CBT in community settings. Trauma Violence Abuse. (2008) 9:214–26. doi: 10.1177/1524838008324336, PMID: 18936280

[30] ISTSS. Posttraumatic stress disorder prevention and treatment guidelines: Methodology and recommendations. Chicago, IL: International Society for Traumatic Stress Studies (2019).

[31] Demerouti E Bakker AB Nachreiner F Schaufeli WB. The job demands-resources model of burnout. J Appl Psychol. (2001) 86:499–512. doi: 10.1037/0021-9010.86.3.499, PMID: 11419809

[32] Schaufeli WB Bakker AB. Job demands, job resources, and their relationship with burnout and engagement: a multi-sample study. J Organ Behav. (2004) 25:293–315. doi: 10.1002/job.248

[33] Bakker AB Demerouti E. Towards a model of work engagement. Career Dev Int. (2008) 13:209–23. https://doi:10.1108/13620430810870476. doi: 10.1108/13620430810870476

[34] Bakker AB Demerouti E Schaufeli WB. Dual processes at work in a call Centre: an application of the job demands-resources model. Eur J Work Organ Psy. (2003) 12:393–417. doi: 10.1080/13594320344000165

[35] Bakker AB Demerouti E Sanz-Vergel A. Job demands–resources theory: Ten years later. Ann Rev Organ Psychol Organiz Behav. (2023) 10:25–53. doi: 10.1146/annurev-orgpsych-120920-053933

[36] Stamm BH. The concise ProQOL manual, vol. 2. Pocatello, ID: ProQOL.org (2010).

[37] Sabo B. Reflecting on the concept of compassion fatigue. Online J Issues Nurs. (2011) 16:1. doi: 10.3912/OJIN.Vol16No01Man01, PMID: 21800932

[38] Figley CR. Compassion fatigue as secondary traumatic stress disorder: an overview In: Figley CR, editor. Compassion fatigue: Coping with secondary traumatic stress disorder in those who treat the traumatized. New York: Brunner-Routledge (1995). 1–20.

[39] Buonomo I Farnese ML Vecina ML Benevene P. Other-focused approach to teaching. The effect of ethical leadership and quiet Ego on work engagement and the mediating role of compassion satisfaction. Front Psychol. (2021) 12:692116. doi: 10.3389/fpsyg.2021.692116, PMID: 34248796 PMC8264287

[40] Chiappo-West G. Compassion satisfaction, burnout, secondary traumatic stress, and work engagement in police officers in Arizona, Doctoral dissertation. Phoenix, AZ: Grand Canyon University (2017).

[41] Audin K Burke J Ivtzan I. Compassion fatigue, compassion satisfaction and work engagement in residential child care. Scott J Resid Child Care. (2018) 17:5–27.

[42] Mason VM Leslie G Clark K Lyons P Walke E Butler C. Compassion fatigue, moral distress, and work engagement in surgical intensive care unit trauma nurses: a pilot study. Dimens Crit Care Nurs. (2014) 33:215–25. doi: 10.1097/DCC.0000000000000056, PMID: 24895952

[43] Kaiser S Patras J Adolfsen F Richardsen AM Martinussen M. Using the job demands–resources model to evaluate work-related outcomes among Norwegian health care workers. SAGE Open. (2020) 2020:436. doi: 10.1177/2158244020947436

[44] Wells-English D Giese J Price J. Compassion fatigue and satisfaction: influence on turnover among oncology nurses at an urban Cancer center. Clin J Oncol Nurs. (2019) 23:487–93. doi: 10.1188/19.CJON.487-493, PMID: 31538984

[45] Fredrickson BL. The role of positive emotions in positive psychology: the broaden-and-build theory of positive emotions. Am Psychol. (2001) 56:218–26. doi: 10.1037/0003-066X.56.3.218, PMID: 11315248 PMC3122271

[46] Hoonakker P Carayon P Korunka C. Using the job-demands-resources model to predict turnover in the information technology workforce: general effects and gender differences. Psihol Obzorja. (2013) 22:51–65. doi: 10.20419/2013.22.373

[47] Russell MB Attoh PA Chase T Gong T Kim J Liggans GL. Examining burnout and the relationships between job characteristics, engagement, and turnover intention among U.S. educators. SAGE Open. (2020) 2020:361. doi: 10.1177/2158244020972361

[48] Schaufeli WB Bakker AB Van Rhenen W. How changes in job demands and resources predict burnout, work engagement and sickness absenteeism. J Organ Behav. (2009) 30:893–917. doi: 10.1002/job.595

[49] Kim YH Kim SR Kim YO Kim JY Kim HK Kim HY. Influence of type D personality on job stress and job satisfaction in clinical nurses: the mediating effects of compassion fatigue, burnout, and compassion satisfaction. J Adv Nurs. (2017) 73:905–16. doi: 10.1111/jan.13177, PMID: 27706839

[50] Voss Horrell SC Holohan DR Didion LM Vance GT. Treating traumatized OEF/OIF veterans: how does trauma treatment affect the clinician? Prof Psychol Res Pract. (2011) 42:79. doi: 10.1037/a0022297

[51] Bakker AB Demerouti E. Multiple levels in job demands-resources theory: implications for employee well-being and performance In: Diener E Oishi S Tay L, editors. Handbook of well-being. Salt Lake City, UT: DEF Publishers (2018)

[52] Lindstrom K Elo AL Skogstad A Dallner M Gamberale F Hottinen V. User’s guide for the QPS Nordic. Copenhagen: General Nordic Questionnaire for Psychological and Social Factors at Work (2000).

[53] Taris TW Leisink PL Schaufeli WB. Applying occupational health theories to educator stress: Contribution of the job demands-resources model. Educator stress: An occupational health perspective, pp. 237–259 (2017).

[54] Rai GS. Turnover intention among long-term care staff: Three possible culprits. Int J Bus Soc Sci. (2015):6, 1–9.

[55] Bonsaksen T Nerdrum P Østertun Geirdal A. Psychological distress and its associations with psychosocial work environment factors in four professional groups: a cross-sectional study. Nurs Health Sci. (2021) 23:698–707. doi: 10.1111/nhs.12856, PMID: 34089225

[56] López-López IM Gómez-Urquiza JL Cañadas GR De la Fuente EI Albendín-García L Cañadas-De la Fuente GA. Prevalence of burnout in mental health nurses and related factors: a systematic review and meta-analysis. Int J Ment Health Nurs. (2019) 28:1035–44. doi: 10.1111/inm.12606, PMID: 31132216

[57] Yanchus NJ Periard D Osatuke K. Further examination of predictors of turnover intention among mental health professionals. J Psychiatr Ment Health Nurs. (2017) 24:41–56. doi: 10.1111/jpm.12354, PMID: 27928857

[58] Miner KN Cortina LM. Observed workplace incivility toward women, perceptions of interpersonal injustice, and observer occupational well-being: differential effects for gender of the observer. Front Psychol. (2016) 7:482. doi: 10.3389/fpsyg.2016.00482 27242558 PMC4868856

[59] Einarsen S Nielsen MB. Workplace bullying as an antecedent of mental health problems: a five-year prospective and representative study. Int Arch Occup Environ Health. (2015) 88:131–42. doi: 10.1007/s00420-014-0944-7, PMID: 24840725

[60] Boudrias V Trépanier S Salin D. A systematic review of research on the longitudinal consequences of workplace bullying and the mechanisms involved. Aggress Violent Behav. (2021) 56:101508. doi: 10.1016/j.avb.2020.101508

[61] Dallner M Elo AL Gamberale F Hottinen V Knardahl S Lindström K. Validation of the general nordic questionnaire (QPSNordic) for psychological and social factors at work. In: Vartiainen M Avallone F Anderson N. Innovative theories, tools, and practices in work and organizational psychology. Boston, MA: Hogrefe and Huber Publishers, Nordic Council of Ministers (2000).

[62] Wännström I Peterson U Asberg M Nygren A Gustavsson JP. Psychometric properties of scales in the general Nordic questionnaire for psychological and social factors at work (QPS): confirmatory factor analysis and prediction of certified long-term sickness absence. Scand J Psychol. (2009) 50:231–44. doi: 10.1111/j.1467-9450.2008.00697.x, PMID: 19037910

[63] Baugerud GA Vangbæk S Melinder AM. Secondary traumatic stress, burnout and compassion satisfaction among Norwegian child protection workers: protective and risk factors. Br J Soc Work. (2018) 48:215–35. doi: 10.1093/bjsw/bcx002

[64] Kaski SS Kinnunen U. Work-related ill-and well-being among Finnish sport coaches: exploring the relationships between job demands, job resources, burnout and work engagement. In J Sports Sci Coach. (2021) 16:262–71. doi: 10.1177/1747954120967794

[65] Bae J Jennings PF Hardeman CP Kim E Lee M Littleton T. Compassion satisfaction among social work practitioners: the role of work–life balance. J Soc Serv Res. (2020) 46:320–30. doi: 10.1080/01488376.2019.1566195

[66] Rugulies R Christensen KB Borritz M Villadsen E Bültmann U Kristensen TS. The contribution of the psychosocial work environment to sickness absence in human service workers: results of a 3-year follow-up study. Work Stress. (2007) 21:293–311. doi: 10.1080/02678370701747549

[67] Thompson I Amatea E Thompson E. Personal and contextual predictors of mental health counselors' compassion fatigue and burnout. J Ment Health Couns. (2014) 36:58–77. doi: 10.17744/mehc.36.1.p61m73373m4617r3

[68] Tripathi N Bharadwaja M. Empowering leadership and psychological health: the mediating role of psychological empowerment. Empl Responsib Rights J. (2020) 32:97–121. doi: 10.1007/s10672-020-09349-9, PMID: 37942772

[69] Schaufeli WB. Applying the job demands-resources model: a ‘how to’ guide to measuring and tackling work engagement and burnout. Organ Dyn. (2017) 46:120–32. doi: 10.1016/j.orgdyn.2017.04.008

[70] Tuckey MR Bakker AB Dollard MF. Empowering leaders optimize working conditions for engagement: a multilevel study. J Occup Health Psychol. (2012) 17:15–27. doi: 10.1037/a0025942, PMID: 22409390

[71] Kruzich JM Mienko JA Courtney ME. Individual and work group influences on turnover intention among public child welfare workers: the effects of work group psychological safety. Child Youth Serv Rev. (2014) 42:20–7. doi: 10.1016/j.childyouth.2014.03.005, PMID: 27409075

[72] Aarons GA Ehrhart MG Farahnak LR. The implementation leadership scale (ILS): development of a brief measure of unit level implementation leadership. Implement Sci. (2014) 9:45. doi: 10.1186/1748-5908-9-45, PMID: 24731295 PMC4022333

[73] Ogden T Bjørnebekk G Kjøbli J Patras J Christiansen T Taraldsen K. Measurement of implementation components ten years after a nationwide introduction of empirically supported programs--a pilot study. Implement Sci. (2012) 7:49. doi: 10.1186/1748-5908-7-49 22651221 PMC3405482

[74] Skogøy BE Sørgaard K Maybery D Ruud T Stavnes K Kufås E. Hospitals implementing changes in law to protect children of ill parents: a cross-sectional study. BMC Health Serv Res. (2018) 18:2. doi: 10.1186/s12913-018-3393-2, PMID: 30081882 PMC6080385

[75] Bronkhorst B Tummers L Steijn B Vijverberg D. Organizational climate and employee mental health outcomes: a systematic review of studies in health care organizations. Health Care Manag Rev. (2015) 40:254–71. doi: 10.1097/HMR.0000000000000026, PMID: 24901297

[76] Miller A Unruh L Wharton T Liu X Zhang N. Individual and organizational factors associated with professional quality of life in Florida fire personnel. J Emerg Manage. (2018) 16:173–82. doi: 10.5055/jem.2018.0366, PMID: 30044490

[77] Huppert FA Whittington JE. Evidence for the independence of positive and negative well-being: implications for quality of life assessment. Br J Health Psychol. (2003) 8:107–22. doi: 10.1348/135910703762879246, PMID: 12643820

[78] Streiner DL. Finding our way: an introduction to path analysis. Can J Psychiatry. (2005) 50:115–22. doi: 10.1177/070674370505000207 15807228

[79] Fixsen D Panzano P Naoom S Blasé K. *Measures of implementation components of the national implementation research network frameworks*. Chapel Hill (2008).

[80] Lauvrud C Nonstad K Palmstierna TO. Occurence of post-traumatic stress symptoms and their relationship to profession-al quality of lilfe (ProQOL) in nursing staff at a forensic psychiatric security unit: a cross sectional study. Health Qual LifeOutc. (2009) 7:31. doi: 10.1186/1477-7525-7-31 PMC2672066 19371413

[81] Bothma CF Roodt G. The validation of the turnover intention scale. SA J Hum Resour Manag. (2013) 11:1–12. doi: 10.4102/sajhrm.v11i1.507

[82] Westfall PH Henning KS. (2013). Understanding advanced statistical methods.Vol. 543. Boca Raton, FL, USA: CRC Press.

[83] R Core Team. R: A language and environment for statistical computing. Vienna, Austria: R Foundation for Statistical Computing (2022).

[84] IBM Corp. IBM SPSS statistics for windows, version 27.0. Armonk, NY: IBM Corp (2020).

[85] Posit Team. RStudio: Integrated development environment for R. Boston, MA: Posit Software (2022).

[86] Rosseel Y. Lavaan: an R package for structural equation modeling. J Stat Softw. (2012) 48:1–36. doi: 10.18637/jss.v048.i02

[87] Kline RB. Principles and practice of structural equation modeling 5th Edn. New York: The Guilford Press, pp. 3–427. (2011).

[88] Korkmaz S Goksuluk D Zararsiz G. MVN: an R package for assessing multivariate normality. R J. (2014) 6:151–62. doi: 10.32614/RJ-2014-031

[89] Fox J Nie Z Byrnes J. *Sem: structural equation Models*. R package version, No. 3, pp. 1–15. (2022). Available at: https://CRAN.R-project.org/package=sem.

[90] Hooper D Coughlan J Mullen M. Structural equation modelling: guidelines for determining model fit. Electron J Bus Res Methods. (2008) 6:53–60.

[91] Hu LT Bentler PM. Cutoff criteria for fit indexes in covariance structure analysis: conventional criteria versus new alternatives. Struct Equ Model. (1999) 6:1–55. doi: 10.1080/10705519909540118, PMID: 36787513

[92] Steiger JH. Understanding the limitations of global fit assessment in structural equation modeling. Pers Individ Diff. (2007) 42:893–8. doi: 10.1016/j.paid.2006.09.017, PMID: 37147182

[93] Preacher KJ Hayes AF. Asymptotic and resampling strategies for assessing and comparing indirect effects in multiple mediator models. Behav Res Methods. (2008) 40:879–91. doi: 10.3758/BRM.40.3.879, PMID: 18697684

[94] Savalei V Rhemtulla M. On obtaining estimates of the fraction of missing information from FIML. Struct Equ Model Multidiscip J. (2012) 19:477–94. doi: 10.1080/10705511.2012.687669

[95] Kenny DA Judd CM. Power anomalies in testing mediation. Psychol Sci. (2014) 25:334–9. doi: 10.1177/0956797613502676, PMID: 24311476

[96] Newsom J. *Practical approaches to dealing with nonnormal and categorical variables*. (2012). Available at: http://web.pdx.edu/~newsomj/semclass/ho_estimate2.pdf.

[97] Norman G. Likert scales, levels of measurement and the “laws” of statistics. Adv Health Sci Educ. (2010) 15:625–32. doi: 10.1007/s10459-010-9222-y, PMID: 20146096

[98] Sullivan GM Artino AR Jr. Analyzing and interpreting data from likert-type scales. J Grad Med Educ. (2013) 5:541–2. doi: 10.4300/JGME-5-4-18, PMID: 24454995 PMC3886444

[99] Johnson DR Creech JC. Ordinal measures in multiple indicator models: a simulation study of categorization error. Am Sociol Rev. (1983) 48:398–407. doi: 10.2307/2095231

[100] Zumbo BD Zimmerman DW. Is the selection of statistical methods governed by level of measurement? Can Psychol. (1993) 34:390–400. doi: 10.1037/h0078865, PMID: 38248460

[101] Wang YA Rhemtulla M. Power analysis for parameter estimation in structural equation modeling: a discussion and tutorial. Adv Methods Pract Psychol Sci. (2021) 4:8253. doi: 10.1177/2515245920918253

[102] Wolf EJ Harrington KM Clark SL Miller MW. Sample size requirements for structural equation models: an evaluation of power, Bias, and solution propriety. Educ Psychol Meas. (2013) 76:913–34. doi: 10.1177/0013164413495237, PMID: 25705052 PMC4334479

[103] Chang WJA Wang YS Huang TC. Work design–related antecedents of turnover intention: a multilevel approach. Hum Resour Manag. (2013) 52:1–26. doi: 10.1002/hrm.21515

[104] Kurtessis JN Eisenberger R Ford MT Buffardi LC Stewart KA Adis CS. Perceived organizational support: a meta-analytic evaluation of organizational support theory. J Manage. (2015) 43:1854–84. doi: 10.1177/0149206315575554

[105] Asfahani AM. The impact of role conflict on turnover intention among faculty members: a moderated mediation model of emotional exhaustion and workplace relational conflict. Front Psychol. (2022) 13:1087947. doi: 10.3389/fpsyg.2022.1087947, PMID: 36619069 PMC9811317

[106] Bourdrias V Trépanier S Salin D. A systematic review of research on the longitudinal consequences of workplace bullying and the mechanisms involved. Aggression and Violent Behavior (2021) 56:101508.

[107] Broeck AV Baillien E Witte HD. Workplace bullying: a perspective from the job demands-resources model. SA J Ind Psychol. (2011) 37:12. doi: 10.4102/sajip.v37i2.879

[108] Brown NC Prashantham BJ Abbott M. Personality, social support and burnout among human service professionals in India. J Community Appl Soc Psychol. (2003) 13:320–4. doi: 10.1002/casp.734, PMID: 27689233

[109] Lobburi P. The influence of organizational and social support on turnover intention in collectivist contexts. J Appl Bus Res. (2012) 28:93–104. doi: 10.19030/jabr.v28i1.6687

[110] Baka L Prusik M Jasielska D. Toward a better understanding of the health impairment process. Types of demand and burnout component matter. Front Psych. (2023) 13:1037053. doi: 10.3389/fpsyt.2022.1037053, PMID: 36699490 PMC9868600

[111] Broetje S Jenny GJ Bauer GF. The key job demands and resources of nursing staff: an integrative review of reviews. Front Psychol. (2020) 11:84. doi: 10.3389/fpsyg.2020.00084, PMID: 32082226 PMC7005600

[112] Zheng G Lyu X Pan L Chen A. The role conflict-burnout-depression link among Chinese female health care and social service providers: the moderating effect of marriage and motherhood. BMC Public Health. (2022) 22:230. doi: 10.1186/s12889-022-12641-y 35120482 PMC8815119

[113] Peter KA Halfens RJG Hahn S Schols JMGA. Factors associated with work-private life conflict and leadership qualities among line managers of health professionals in Swiss acute and rehabilitation hospitals - a cross-sectional study. BMC Health Serv Res. (2021) 21:81. doi: 10.1186/s12913-021-06092-1 33482808 PMC7821733

[114] Vadvilavicius T Stelmokiene A. Evidence-based practices that deal with work-family conflict and enrichment: Sysematic literature review. Verslas. (2020) 21:820–6. doi: 10.3846/btp.2020.12252

[115] Podsakoff NP LePine JA LePine MA. Differential challenge stressor-hindrance stressor relationships with job attitudes, turnover intentions, turnover, and withdrawal behavior: a meta-analysis. J Appl Psychol. (2007) 92:438. doi: 10.1037/0021-9010.92.2.438, PMID: 17371090

[116] Dodanwala TC Santoso DS Shrestha P. The mediating role of work–family conflict on role overload and job stress linkage. Built Environ. Project Asset Manage. (2022) 12:924–39. doi: 10.1108/BEPAM-12-2021-0153

[117] Nielsen K Nielsen M Ogbonnaya C Känsälä M Saari E Isaksson K. Workplace resources to improve both employee well-being and performance: a systematic review and meta-analysis. Work Stress. (2017) 31:101–20. doi: 10.1080/02678373.2017.1304463, PMID: 30480770

[118] Niinihuhta M Terkamo-Moisio A Kvist T Häggman-Laitila A. A comprehensive evaluation of factors affecting nurse leaders’ work-related well-being. Leadersh Health Serv. (2022) 35:460–74. doi: 10.1108/LHS-12-2021-0098, PMID: 35543569 PMC9590637

[119] Indregard AMR Ulleberg P Knardahl S Nielsen MB. Emotional dissonance and sickness absence among employees working with customers and clients: a moderated mediation model via exhaustion and human resource primacy. Front Psychol. (2018) 9:436. doi: 10.3389/fpsyg.2018.00436 29670556 PMC5893769

[120] Yıldırım N Coşkun H Polat Ş. The relationship between psychological capital and the occupational psychologic risks of nurses: the mediation role of compassion satisfaction. J Nurs Scholarsh. (2021) 53:115–25. doi: 10.1111/jnu.12607, PMID: 33146952

[121] Arnoux-Nicolas C Sovet L Lhotellier L Di Fabio A Bernaud JL. Perceived work conditions and turnover intentions: the mediating role of meaning of work. Front Psychol. (2016) 7:704. doi: 10.3389/fpsyg.2016.00704, PMID: 27242616 PMC4863887

[122] Tremblay MA Messervey DL. (2011). The Job Demands-Resources Model: Further Evidence for the Buffering Effect of Personal Resources. Sa Journal of Industrial Psychology (2011):10–19.

[123] Hopstaken JF van der Linden D Bakker AB Kompier MA. (2015). A multifaceted investigation of the link between mental fatigue and task disengagement. Psychophysiology (2015):305–315. doi: 10.1111/psyp.12339 25263028

[124] Conway JM Lance CE. What reviewers should expect from authors regarding common method bias in organizational research. J Bus Psychol. (2010) 25:325–34. doi: 10.1007/s10869-010-9181-6, PMID: 37842826

[125] Stage FK Carter HC Nora A. Path analysis: an introduction and analysis of a decade of research. J Educ Res. (2004) 98:5–13. doi: 10.3200/JOER.98.1.5-13, PMID: 38228564

[126] Hawley KM Cook JR Jensen-Doss A. Do noncontingent incentives increase survey response rates among mental health providers? A randomized trial comparison. Admin Pol Ment Health. (2009) 36:343–8. doi: 10.1007/s10488-009-0225-z, PMID: 19421851 PMC2715443

[127] Newman DA. (2014). Missing data: Five practical guidelines. Organizational Research Methods (2014):372–411. doi: 10.1177/1094428114548590

[128] Statistics Norway. (2022). Available at: https://www.ssb.no/statbank/table/09550 (Accessed April 7, 2022).

