# Supplementary material for: Exploring key job demands and resources in Norwegian child mental health services: a cross-sectional study of associations with and relationship between compassion satisfaction, burnout, secondary traumatic stress and turnover intention
Source: Front Public Health. 2024 Mar 6;12:1304345. doi: 10.3389/fpubh.2024.1304345 (PMC10961911; doi:10.3389/fpubh.2024.1304345)
Supplement: Supplementary file 1 [file Table_1.DOCX]

Supplementary Table 1. Pearson`s correlation analysis between exogenous variables

| **18** | 0.02 | -0.15* | -0.18** | -0.09 | 0.49*** | -0.180** | 0.37*** | -0.23*** | -0.10 | -0.33*** | -0.21*** | -0.22*** | -0.03 | -0.19** | 0.11 | -0.19** | -0.20** | - |
| --- | --- | --- | --- | --- | --- | --- | --- | --- | --- | --- | --- | --- | --- | --- | --- | --- | --- | --- |
| **17** | 0.01 | 0.33*** | 0.61*** | 0.21** | -0.18** | 0.33*** | -0.38*** | 0.39*** | 0.28*** | 0.47*** | 0.42*** | 0.66*** | 0.19** | 0.55*** | -0.15* | 0.71*** | — | - |
| **16** | 0.12 | 0.32*** | 0.60*** | 0.24*** | -0.13* | 0.30*** | -0.33*** | 0.46*** | 0.34*** | 0.42*** | 0.44*** | 0.68*** | 0.20** | 0.58*** | -0.04 | — |  | - |
| **15** | -0.08 | -0.14* | -0.12 | -0.17** | 0.15* | -0.25*** | 0.24*** | -0.15* | 0.04 | -0.18** | -0.25*** | -0.19** | -0.004 | -0.18** | — |  |  | - |
| **14** | 0.14* | 0.27*** | 0.43*** | 0.28*** | -0.12 | 0.36*** | -0.33*** | 0.46*** | 0.33*** | 0.50*** | 0.79*** | 0.79*** | 0.70*** | — |  |  |  | - |
| **13** | 0.16* | 0.04 | 0.07 | 0.05 | 0.003 | 0.05 | -0.055 | 0.21*** | 0.13* | 0.16** | 0.30*** | 0.20** | — |  |  |  |  | - |
| **12** | 0.06 | 0.38*** | 0.59*** | 0.31*** | -0.16** | 0.40*** | -0.39*** | 0.40*** | 0.32*** | 0.50*** | 0.63*** | — |  |  |  |  |  | - |
| **11** | 0.09 | 0.19** | 0.34*** | 0.32*** | -0.14* | 0.41*** | -0.32*** | 0.46*** | 0.33*** | 0.52*** | — |  |  |  |  |  |  | - |
| **10** | -0.09 | 0.24*** | 0.39*** | 0.23*** | -0.22*** | 0.51*** | -0.42*** | 0.40*** | 0.30*** | — |  |  |  |  |  |  |  | - |
| **9** | 0.001 | 0.22*** | 0.25*** | 0.25*** | -0.04 | 0.30*** | -0.31*** | 0.40*** | — |  |  |  |  |  |  |  |  | - |
| **8** | 0.06 | 0.26*** | 0.25*** | 0.33*** | -0.20** | 0.39*** | -0.36*** | — |  |  |  |  |  |  |  |  |  | - |
| **7** | -0.02 | -0.33*** | -0.29*** | -0.25*** | 0.38*** | -0.32*** | — |  |  |  |  |  |  |  |  |  |  | - |
| **6** | -0.02 | 0.19** | 0.28*** | 0.25*** | 0.012 | — |  |  |  |  |  |  |  |  |  |  |  | - |
| **5** | 0.04 | -0.19 | - 0.20** | -0.08 | — |  |  |  |  |  |  |  |  |  |  |  |  | - |
| **4** | 0.36*** | 0.39*** | 0.44*** | — |  |  |  |  |  |  |  |  |  |  |  |  |  | - |
| **3** | 0.19** | 0.49*** | — |  |  |  |  |  |  |  |  |  |  |  |  |  |  | - |
| **2** | 0.29*** | — |  |  |  |  |  |  |  |  |  |  |  |  |  |  |  | - |
| **1** | — |  |  |  |  |  |  |  |  |  |  |  |  |  |  |  |  | - |
| Variable | **1. Facilitative Administration** | **2. System Intervention** | **3. Implementation Leadership** | **4. Implementation Climate** | **5. Quantitative job demands** | **6. Positive Challenges** | **7. Role Conflict** | **8. Control of Decision** | **9. Predictability** | **10. Mastery of Work** | **11. Support CoWorkers** | **12. Support Supervisor** | **13. Support Famliy** | **14. total Support** | **15. Bullying Harassment** | **16. Empowering Leadership** | **17. Human resource primacy** | **18. Work-life interference** |

*, **, *** Significant at the 0.05, 0.01, and 0.001 level, respectively.
